# Sintilimab-induced intestinal obstruction and hemorrhage of the digestive tract: a case report

**DOI:** 10.3389/fimmu.2025.1590714

**Published:** 2025-07-31

**Authors:** Qian Hao, Wenwen Zhu, Qingqing Guo, Xiuxiu Lu, Shijun Zhang, Jianjun Yang, Zhonghua Song

**Affiliations:** ^1^ Department of General Practice, Shandong Provincial Third Hospital, Shandong, Jinan, China; ^2^ Department of Gastroenterology, Central Hospital Affiliated to Shandong First Medical University, Shandong, Jinan, China; ^3^ Department of Infectious Diseases, Peking University Healthcare Lu Zhong Hospital, Shandong, Zibo, China

**Keywords:** sintilimab, immune checkpoint inhibitor, hemorrhage of the digestive tract, intestinal obstruction, immune-related adverse event

## Abstract

Immune checkpoint inhibitors (ICIs) have become an important part of malignant tumor therapy. However, some adverse reactions follow ICIs therapy. The incidence of immune-mediated colitis (IMC) has also increased. Here, the case of a 67-year-old male patient with stage IVB esophagogastric junction squamous cell carcinoma after chemotherapy, targeted therapy and immunotherapy is presented. This patient underwent treatment with six courses of protein-bound paclitaxel + cis-platinum+ sintilimab, followed by maintenance therapy with sintilimab alone. The patient developed intestinal obstruction, abdominal pain, hemorrhage of the lower digestive tract and other discomfort and was diagnosed with multiple jejunal ulcers via colonoscopy and pathological biopsy. The aims of presenting this case report are to improve clinicians’ understanding of and ability to treat sintilimab-related adverse reactions and to more scientifically and rationally administer sintilimab for the treatment of malignant tumors.

## 1 Introduction

Tumor immunotherapy has been another breakthrough in tumor treatment. Immune checkpoint inhibitors (ICIs) have been widely used to treat melanoma, non-small cell lung cancer, liver cancer, stomach cancer and other malignant tumors. ICIs therapy mainly restores and improves the ability of effector T lymphocytes to specifically recognize and kill cancer cells through the binding of ICIs to immune checkpoints, thus strengthening the body’s antitumor immune response (1). ICIs not only strengthen the tumor-specific immune response but also nonspecifically activate the immune system, leading to the destruction of immune homeostasis, which can result in immune-related adverse effects (irAEs) (2–4). The exact pathophysiological mechanism of irAEs is still unclear, but the findings of relevant studies of irAEs suggest that T-cell, B-cell, antibody, and cytokine responses may be involved (1, 2, 5). Sintilimab is a recombinant human immunoglobulin G anti-programmed cell death 1 (PD-1) monoclonal antibody. By binding to PD-1 and blocking the binding of PD-1 to PD-L1 and PD-L2, sintilimab injection can relieve the immunosuppressive effect, activate the function of T cells, strengthen the immune surveillance and ability of T cells to kill tumors, and generate a tumor immune response (6, 7). Some studies have also shown that the risk of developing irAEs may be associated with factors such as germline genetic factors and the composition of the host microbiota (8, 9). In recent years, the incidence of immune-mediated colitis (IMC) has increased. While there have been previous reports of IMC induced by PD-1 inhibitors, there have been no documented cases of IMC, intestinal obstruction or hemorrhage of the digestive tract caused by sintilimab simultaneously.

In this case report, the diagnosis and treatment processes of a patient with a malignant tumor at the esophagogastric junction who developed IMC, intestinal obstruction and hemorrhage of the digestive tract after undergoing chemotherapy and anti-PD-1 immunotherapy are presented. The aims of presenting this case report are to improve clinicians’ understanding of and ability to manage the adverse reactions associated with sintilimab and to contribute to the knowledge base on the adverse effects of ICIs.

## 2 Case presentation

A 67-year-old male was diagnosed with carcinoma of the esophagogastric junction (cT3N1M1 stage IVB). Pathological analysis revealed undifferentiated squamous cells. Immunohistochemical staining results were as follows: CK5/6 (+), P40 (+), CK (+), Ki67 (80%), P53 (80%), CK (+), and LCA (-). Immunohistochemical staining results were as follows: CK (+), CK7 (+), TTF1 (+), Napsin A (part+), Vim (+), Syn (-), CgA (-), CD56 (-), P40 (-), CK5/6 (-), and Ki67 (50%+). From the time of diagnosis (from September 2023 to January 2024), the patient received 6 cycles of sintilimab 200 mg (Xinda Pharmaceutical (Suzhou) Co., Ltd.), paclitaxel (albumin–bound) 200 mg (Qilu Pharmaceutical Co., Ltd.) and cisplatin 100 mg (Qilu Pharmaceutical Co., Ltd.). During the fourth cycle of treatment, the patient manifested diarrhea symptoms. Compared with the baseline period, the frequency of bowel movements increased to 4 to 6 times per day, with a small amount of stool and unformed consistency. A test for fecal occult blood was initially positive but became negative upon reexamination. Comprehensive tests for *Vibrio cholerae* and stool culture failed to detect any pathogens. After the administration of montmorillonite powder and loperamide, the symptoms were completely alleviated.

Next, the patient received 2 cycles of sintilimab (200 mg) from March 2024 to April 2024. Approximately 4 days later, the patient presented with symptoms of abdominal pain. Four days after the last discharge, the patient experienced a change in bowel habits, with unformed and scant stools, abdominal pain, and cessation of defecation and flatus. The patient sought medical attention at a community clinic and was administered two intramuscular injections of anisodamine; however, the symptoms failed to remit significantly. The patient presented to our hospital for treatment on the same day (April 16, 2024).

**Figure 1:**
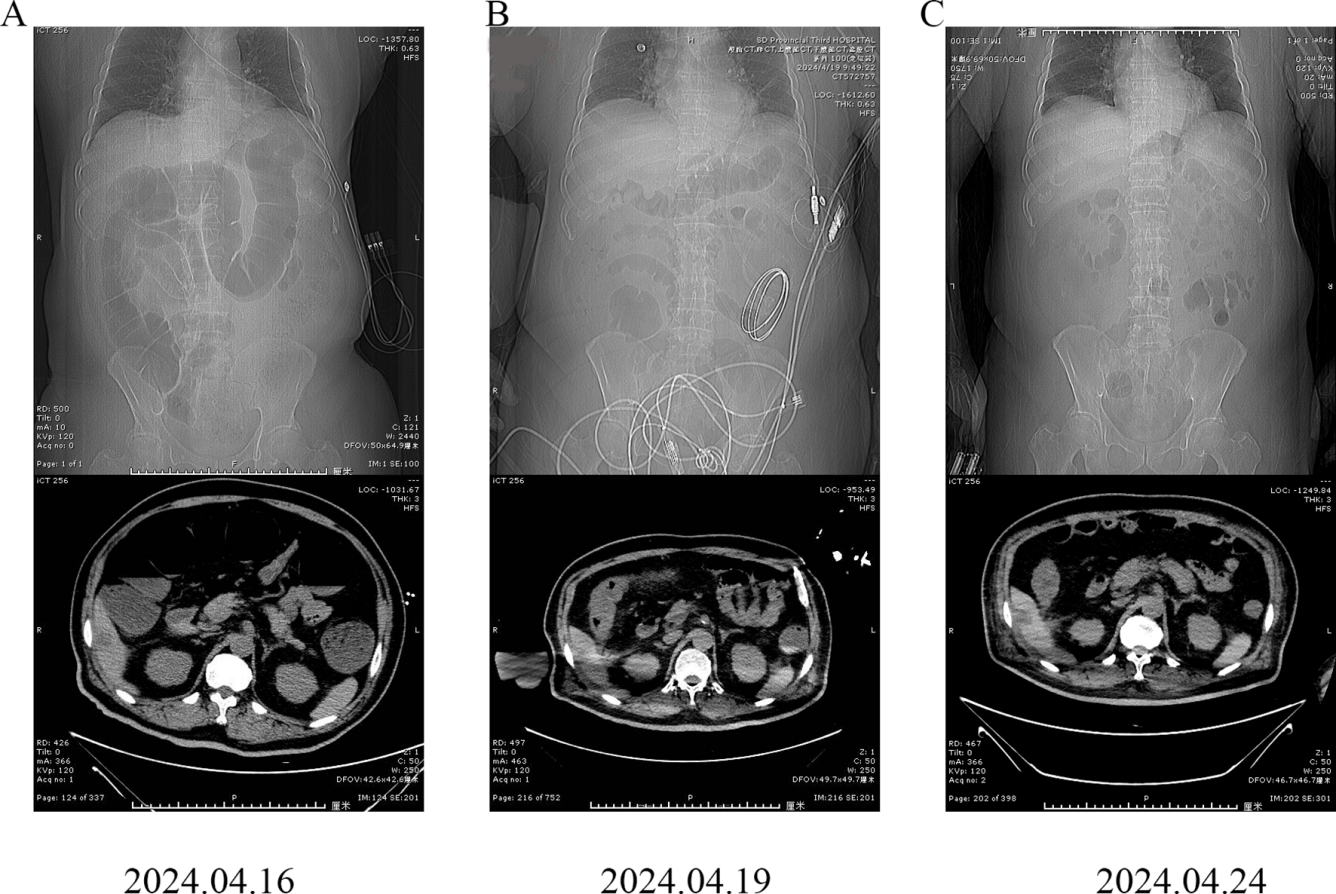
Abdominal computed tomography (CT) images of the patient. **(A)** April 16, 2024; **(B)** April 19, 2024; **(C)** April 24, 2024.

An abdominal computed tomography (CT) scan revealed intestinal obstruction. Multiple dilated intestinal areas were observed, and multiple air–fluid levels were identified (**Figure 1A**). Moreover, the patient presented with electrolyte imbalance, hyperkalemia, peritonitis, and acute renal failure. The patient’s condition was critical, and he was transferred to the intensive care unit (ICU) on April 17, 2024. Symptomatic treatments such as enemas for defecation, meropenem for anti-infection, potassium level reduction, fluid infusion, acid and enzyme suppression, and maintaining homeostasis were given. IrAEs cannot be ruled out, and methylprednisolone treatment is used. The patient subsequently developed acute myocardial infarction and acute pancreatitis. Drug treatments such as kidney-protective agents, myocardial nutritional agents, and pancreatic enzyme secretion inhibitors were given. Compared with before, the symptoms of abdominal pain were relieved. Subsequently, CT re-examinations were conducted on April 19, 2024 and April 24, 2024 (**Figures 1B, C**). There was no swelling of the pancreas, no obvious exudate around the pancreas, and a significant improvement in the degree of intestinal obstruction.

On April 26, 2024, the patient was transferred back to the general ward for further treatment. Starting on April 28, 2024, the patient experienced an increased frequency of defecation, with 7 bowel movements per day, approximately 100–150 ml each time, and the color was dark red. Bowel sounds were inaudible. The test for fecal occult blood was positive, indicating active bleeding. Successively, a thrombin powder enema, hemocoagulase injection, posterior pituitary hormone injection, and somatostatin injection were administered for hemostasis, and leukocyte-free suspended red blood cells were transfused to correct anemia. Nevertheless, the symptoms of active bleeding did not significantly improve.

**Figure 2:**
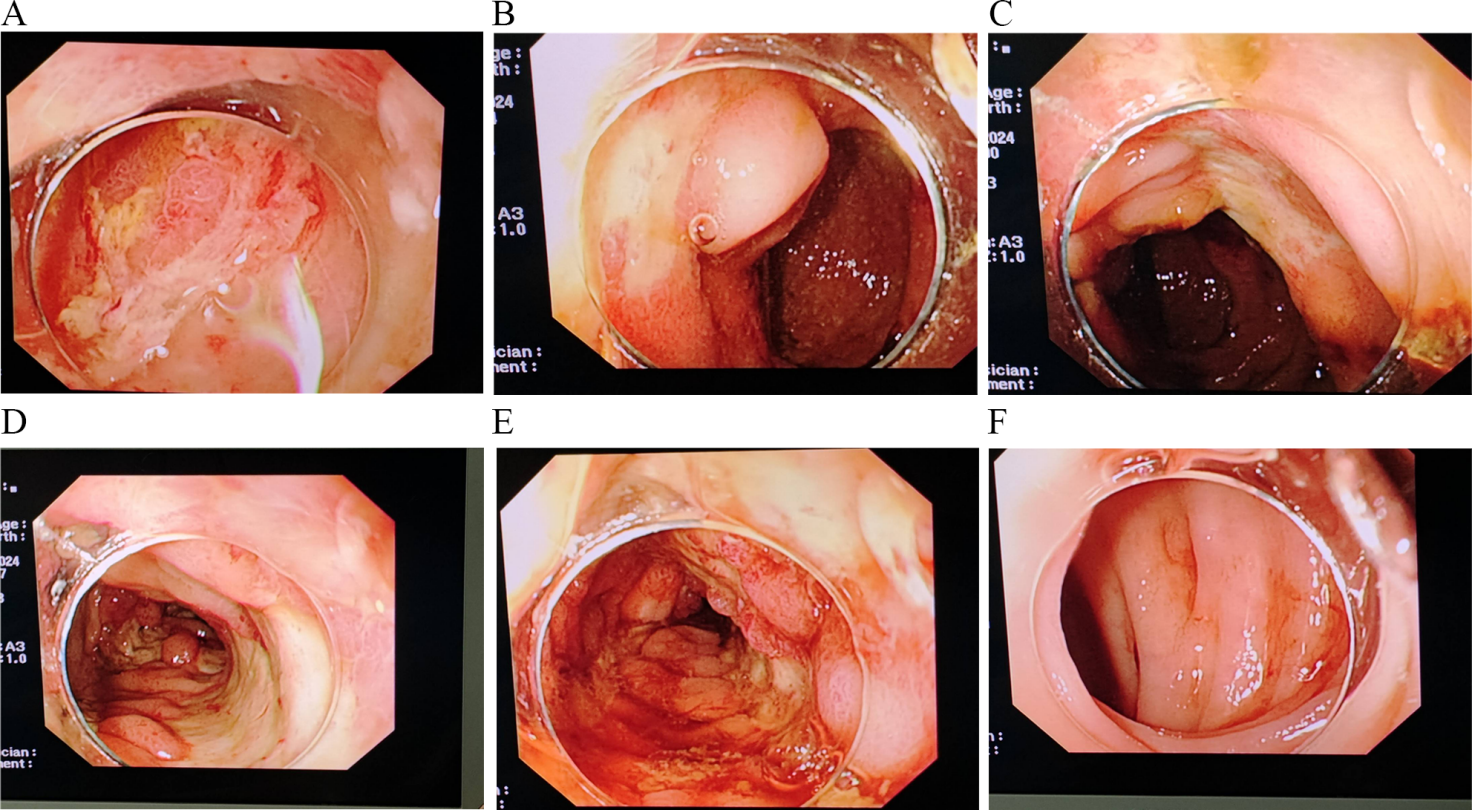
Significant congestion and swelling of the mucosa of the ileocecal region, ascending colon, transverse colon, and descending colon **(A–F)** are observed, with scattered ulcers of varying sizes, some of which are longitudinal and covered with a white coating at the base.

**Figure 3:**
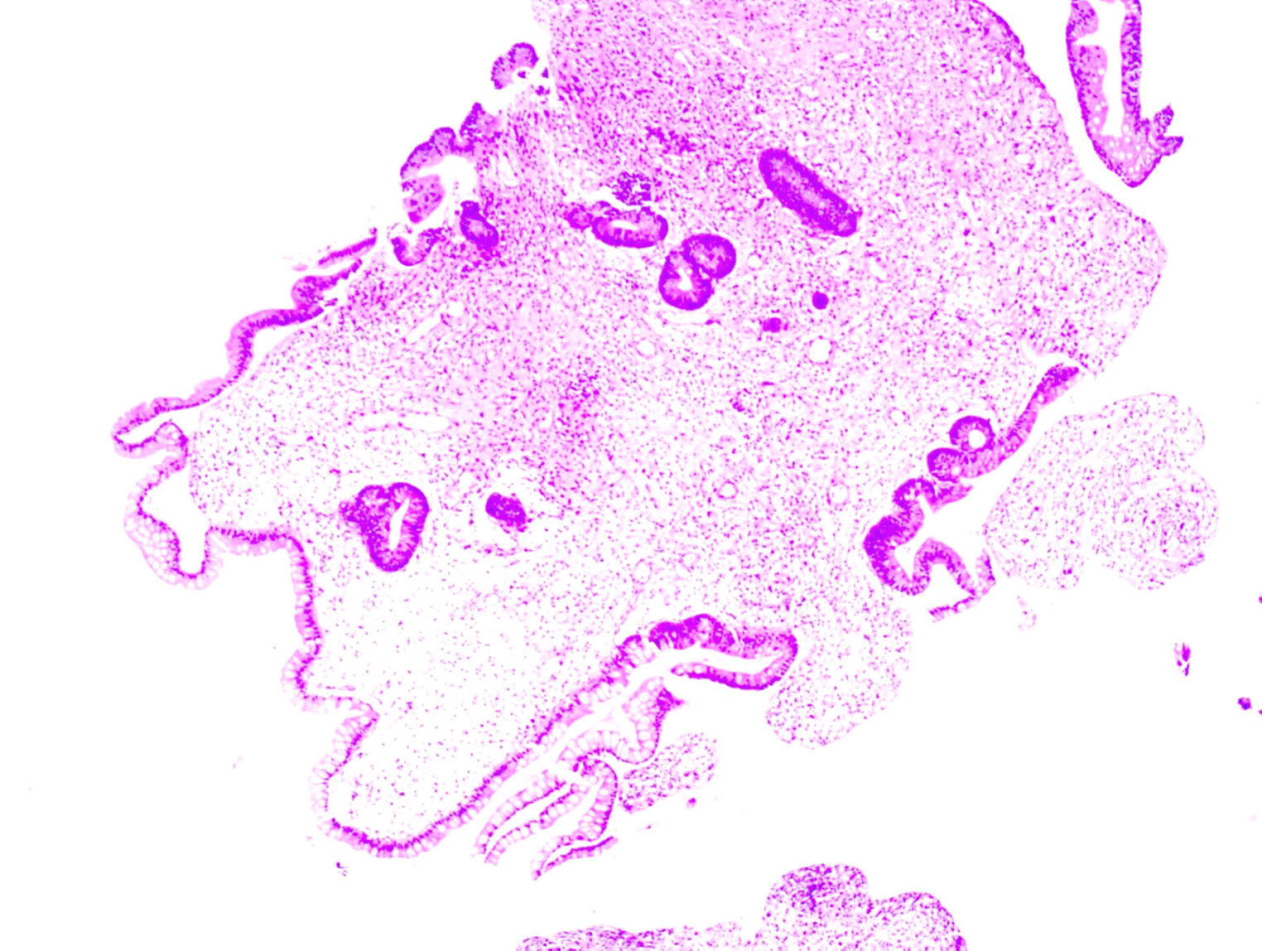
Pathological image of the biopsy specimen, showing chronic active inflammation of the mucosa with ulcer formation; a significant reduction in the number of crypts; atrophy and flattening of the remaining crypts; aggregation of lymphocytes, plasma cells, and granulocytes in the lamina propria; and atypical changes in some glands.

On April 29, 2024, the patient became irritable, and the systolic blood pressure dropped to its lowest level at 80 mmHg. After emergency blood transfusion, the patient was transferred to the ICU again. Red blood cells were transfused intermittently to correct anemia. After the patient’s condition stabilized, a colonoscopy was performed, which revealed significant mucosal congestion and swelling in the terminal ileum, with scattered ulcers of varying sizes covered with a white coating at the base. Significant congestion and swelling of the mucosa of the ileocecal region, ascending colon, transverse colon, and descending colon were also observed, with scattered ulcers of varying sizes, some of which were longitudinal and covered with a white coating at the base (**Figure 2**). The adhesion of the remaining sigmoid and rectum was smooth, the veins were clear, and the semilunar folds were smooth. No decay or new organisms were observed, and the anal canal mucosa was hyperemic and edematous. Pathology of the biopsy sample revealed chronic active inflammation of the mucosa with ulcer formation; a significant reduction in the number of crypts; atrophy and flattening of the remaining crypts; aggregation of lymphocytes, plasma cells, and granulocytes in the lamina propria; and atypical changes in some glands (**Figure 3**). Fecal calprotectin quantification: 132 μg/g. Following multidisciplinary consultations by the departments of Gastroenterology, Oncology, Minimally Invasive Intervention, General Medicine, Gastrointestinal Surgery, Nephrology, Rheumatology and Immunology, and Clinical Pharmacy, the diagnosis of “IMC” was considered. Referring to the progress in the diagnosis and treatment of ICIs (10, 11), the patient’s effect of hormone application was not good, and infliximab was added. Methylprednisolone(80 mg, once daily) and infliximab (5 mg/kg) were administered. On April 30, 2024, superior mesenteric artery angiography was performed, and a catheter was indwelled for local infusion of vasoconstrictive and hemostatic drugs. After the application of the above regimens, the patient’s hematochezia decreased compared with that before; however, intermittent dark red bloody stools still occurred. Intermittent blood transfusion was given to correct anemia, and other treatments were given.

On May 10, 2024, the patient was transferred back to the general ward for treatment. There was still intermittent bleeding, with 4 to 5 episodes of bloody stool per day. During this period, the lowest hemoglobin level was 49.00 g/L. Intermittent infusions of leukoreduced red blood cells, virus-inactivated plasma, and plasma cryoprecipitate were administered, and terlipressin was intermittently infused. On May 14, vedolizumab (300 mg) was given for symptomatic treatment, and the patient’s gastrointestinal bleeding gradually stabilized. On the night of May 25, the patient again had bloody stool, with a total volume of approximately 700 ml. Symptomatic treatment, such as blood transfusion, was given. During the medication cycle, vedolizumab (300 mg) was administered again, and the amount of bleeding decreased.

**Figure 4:**
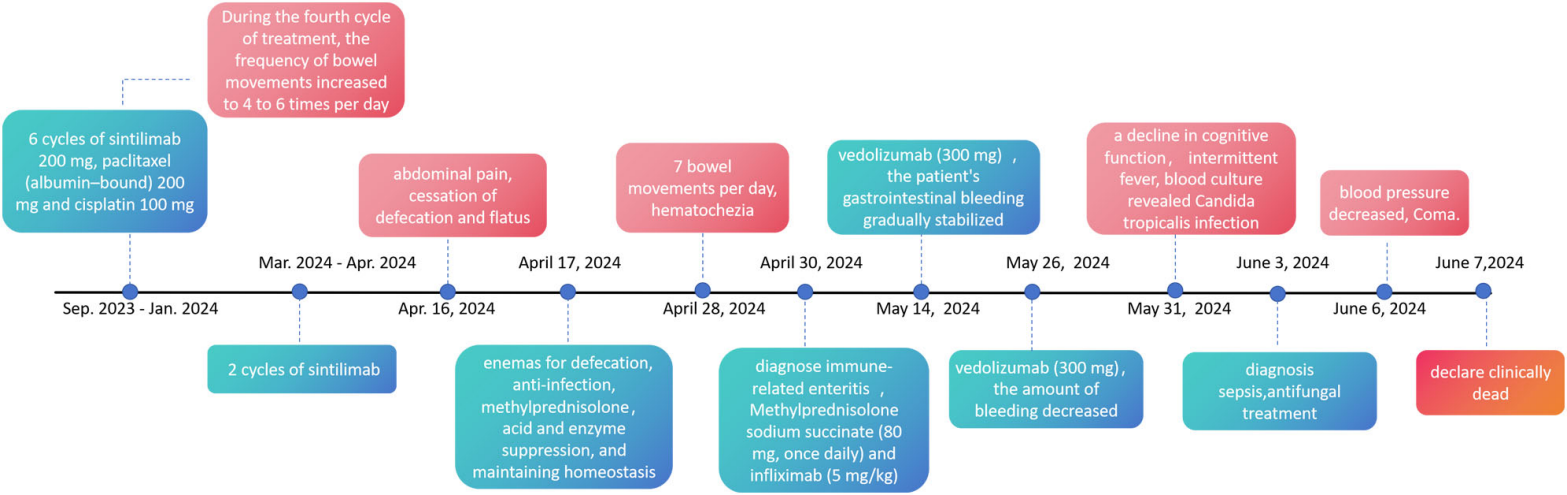
The primary time nodes of patient’s disease occurrence, irAEs onset, development, diagnosis, interventions, treatment, and follow-up.

Beginning on May 31, 2024, the patient began to exhibit a decline in cognitive function, increased sleepiness, and a poor mental state. Laboratory tests revealed a G test result of 137.18 pg/ml; blood culture revealed *Candida tropicalis* infection, which was resistant to fluconazole. By June 3, 2024, the patient experienced drowsiness and intermittent fever, leading to a diagnosis of “sepsis”. The treatment was adjusted to include caspofungin for antifungal therapy. Nevertheless, the patient continued to have intermittent fever, with the highest recorded temperature reaching 38.5 degrees Celsius. On June 6, the patient’s blood pressure decreased. The family chose not to transfer the patient to the ICU or to perform chest compressions or end-of-life resuscitation. The patient was declared clinically dead on June 7, 2024. **Figure 4** illustrates the primary time nodes of patient’s disease occurrence, irAEs onset, development, diagnosis, interventions, treatment, and follow-up.

## 3 Discussion

The discovery of ICIs represents a significant medical advancement in the treatment of cancer, and the benefits they provide to cancer patients are undeniable. However, it is also crucial to pay attention to the adverse reactions that may arise during treatment.

Notably, the patient’s gastrointestinal reactions appeared sometime after the initiation of sintilimab treatment rather than at the beginning. This appearance of reactions after ICIs initiation suggests that when immunotherapeutic drugs such as sintilimab are used, the patient’s condition should be monitored throughout the entire treatment process, and regular follow-up should be conducted to promptly identify and manage potential side effects. Furthermore, for patients with a history of gastrointestinal diseases, extra caution should be exercised when using such drugs, and increased frequency of monitoring and in-depth evaluation should be implemented during treatment.

Here, we present the case of a patient with a tumor at the gastroesophageal junction who underwent treatment with sintilimab, paclitaxel (albumin-bound), and cisplatin. The patient subsequently experienced symptoms ranging from intestinal obstruction to hemorrhage of the digestive tract. During the diagnostic process, we ruled out drug-related causes and intestinal symptoms induced by infections through detailed medical history-taking, stool routine tests, and stool cultures. Laboratory tests excluded metabolic disorders such as diabetes and thyroid dysfunction as potential causes of the intestinal symptoms. Additionally, colonoscopy helped eliminate diagnoses such as ischemic colitis, Crohn’s disease, ulcerative colitis, intestinal metastasis, and primary tumor infiltration. We believe that the adverse reactions induced by the ICIs led to colitis and gastrointestinal bleeding. The patient was then administered corticosteroids, infliximab, and vedolizumab, along with octreotide and esomeprazole. Although the patient ultimately succumbed to septic shock, in this case, we observed that early use of glucocorticoids may be effective. The hemostatic effect was most pronounced after the application of infliximab and vedolizumab, which significantly reduced the production of bloody stools, which benefitted the patient and minimized the risk of intestinal toxicity. In a review of the literature, we also found that vedolizumab is associated with a risk of fungal infections. However, it is difficult to conclusively determine whether the patient’s final fungal infection was related to vedolizumab use. Owing to the lack of data from large-scale studies, further accumulation of clinical trial and real-world data is still necessary. A PubMed search with the keywords “colitis” and “sintilimab” showed three case reports on colitis due to sintilimab (**Table 1**) (12–14). There are a total of 4 patients except for this article. Among them, 2 cases completed the anti-tumor treatment. One case did not stop the treatment. After the improvement of IMC, immune-related ureteritis occurred subsequently. In terms of treatment, all patients received hormone therapy. Two of the patients received vedolizumab treatment, and the other two only received hormone therapy.

**Table 1:** Summary of immune-mediated colitis caused by sintilimab.

Reference	Underlying disease	Intestinal lesion	Dose of sintilimab	Onset time	Whether to perform a colonoscopy	Treatment of esophageal lesion	Time of therapy	Outcome and follow-up
(12)	Primary liver cancer	IMC and inflammatory intestinal obstruction	100 mg	Day 5	No	Glucocorticoid, somatostatin, and cefoperazone sodium/tazobactam	11days	Discharged with recovery
(13)	Esophageal squamous cell carcinoma	IMC	Not described	Day 30	Yes	Mesalazine, somatostatin, cefmenoxime, metronidazole, methylprednisolone, vedolizumab	14 days	Discharged with recovery, not continuing anti-tumor
(13)	Lung adenocarcinoma	IMC	Not described	Day 20	Yes	Somatostatin, cefmenoxime, metronidazole, methylprednisolone, vedolizumab	28days	Discharged with recovery, not continuing anti-tumor
(14)	Pancreatic cancer	IMC	200mg	Day 60	No	metronidazole	5days	IMC was recovered, but PD-1 induced immune ureteritis was occurred. Death of cachexia and depression
Our case	Carcinoma of the esophagogastric junction	IMC, hemorrhage of the digestive tract, intestinal obstruction and intestinal obstruction	200mg	Day 168	Yes	Octreotide, esomeprazole, meropenem, methylprednisolone, infliximab, vedolizumab.	50 days	Bleeding decreased, and dead of fungal infection

In summary, as the use of ICIs in cancer treatment becomes increasingly prevalent, the incidence of irAEs is also increasing. Immune-related colitis, a common irAEs, can lead to severe complications such as intestinal obstruction and perforation. It is crucial to remain vigilant when encountering patients with similar symptoms. A thorough review of the patient’s medication history and past medical history and repeated routine stool tests and occult blood tests are essential. Early completion of digestive endoscopy to exclude other causes of colonic ulcers or inflammation is necessary. Early identification of these conditions is of paramount importance.

These case also underscores the importance of personalized treatment. Patients may exhibit varying responses to immunotherapeutic drugs; hence, it is imperative to tailor individualized treatment plans on the basis of the specific circumstances of each patient and to continuously evaluate and adjust the treatment regimen throughout therapy. Personalized treatment can maximize therapeutic efficacy while minimizing the incidence of adverse effects.

## Data Availability

The original contributions presented in the study are included in the article/supplementary material. Further inquiries can be directed to the corresponding author.
